# Bibliometric analysis and key messages of integrating Chinese and Western Medicine for COVID-19

**DOI:** 10.1016/j.heliyon.2024.e27293

**Published:** 2024-03-08

**Authors:** Meijiao Du, Hongkai Li, Huijuan Guo, Xiaowen Zhang, Hongguo Rong, Xuezeng Hao

**Affiliations:** a School of Traditional Chinese Medicine, Beijing University of Chinese Medicine, Beijing 100029, China; b Clinical Medical College of China-Japan Friendship Hospital, Beijing University of Chinese Medicine, Beijing, 100029, China; c Research Institute of Beijing Tongrentang Co. Ltd., Beijing, 100079, China; d Beijing Tongrentang Technology Development Co. Ltd., Beijing, 100079, China; e Institute for Excellence in Evidence-Based Chinese Medicine, Beijing University of Chinese Medicine, Beijing, 100029, China; f Dongzhimen Hospital, Beijing University of Chinese Medicine, Beijing, 100700, China

**Keywords:** Bibliometric analysis, COVID-19, Treatment, Integrating Chinese and Western medicine

## Abstract

**Background:**

The coronavirus disease 2019 (COVID-19) has been a global pandemic since it broke out, and integrated Chinese and Western medicine (ICWM) has played an important role in the prevention and treatment of COVID-19. We aimed to analyze the published literature on ICWM for COVID-19 at home and abroad, and compare their differences on hotspots and research fronts.

**Methods:**

Publications before Oct 31, 2022 were retrieved from the Web of Science core database (WOS), PubMed, China National Knowledge Infrastructure database (CNKI), Wanfang Data Knowledge Service Platform (Wanfang), China Science and Technology Journal Database (VIP), China Biology Medicine disc (CBM), CiteSpace and VOSviewer to summarize the basic characteristics of publications, countries, institutions, keywords, and citations.

**Results:**

We included 580 English papers and 1727 Chinese papers in this study. The development trends in China and other countries are relatively asynchronous and show a smooth growth trend for the future. The most productive countries were China, India, and the United States, while the most productive domestic research institution was the Beijing University of Chinese Medicine. The clustering analysis of high-frequency keywords showed that Chinese literature focused on clinical studies of ICWM for COVID-19, such as retrospective studies, clinical features, and traditional Chinese medicine syndrome analysis, while English literature focused on therapeutic mechanism studies and evidence-based medicine studies, such as systematic reviews and meta-analysis, and both of them paid attention to network pharmacological research and *Qingfei Paidu Decoction*. Sorting out the top 10 highly cited articles, Huang CL's article published in Lancet in 2020 was regarded as a cornerstone in the field.

**Conclusion:**

The treatment of COVID-19 by ICWM has become a worldwide research hotspot. Although there are differences in the specific contents among countries, the development trend of research types to the mechanism of action, and the development trend of research contents to the recovery period treatment and the prevention of COVID-19 by ICWM are consistent.

## 1 Introduction

Coronavirus disease 2019 (COVID-19) is an infectious disease caused by the SARS-CoV-2 virus, which has been a global pandemic since it broke out in December 2019. People with COVID-19 have had the main clinical manifestations of fever, cough, shortness of breath and muscular aches [1]. According to the World Health Organization (WHO) Outbreak Report data, until the end of October 31, 2022, there have been 627 million confirmed cases of COVID-19 reported globally and 6570 thousand cumulative deaths [2]. The COVID-19 pandemic has exposed the health effects of longstanding social inequities, and also shaken up the socio-economic order on a global scale [3]. Economies were hit top-down and bottom-up while businesses and individuals alike endured significant changes that altered national and international supply and demand trends for products and services [4]. Therefore, effective prevention and treatment of COVID-19 is a very urgent task.

The first version of a living evidence-based guideline of Integrated Chinese and Western medicine (ICWM) for COVID-19, based on Western medicine support treatment, recommended traditional Chinese medicine (TCM) prescription granules/decoction, Chinese patent medicines and Chinese herbal injection for the treatment of mild and moderate COVID-19 [5]. In the absence of effective antiviral drugs, ICWM has played an important role in the prevention and treatment of COVID-19 [6,7]. Since the outbreak, China proposed and applied the treatment of ICWM, which attracted increasing attention from the international community, and relevant studies have been increasingly extensive [8]. With the COVID-19 pandemic having lasted more than 3 years, post-acute sequelae of SARS-CoV-2 (PASC), also known as long coronavirus disease 2019 (Long COVID), represents a growing concern in public healthcare [9]. Long COVID has been linked to more than 200 symptoms, from brain fog or fatigue that makes it difficult to work, to debilitating pain and muscle weakness [10]. There has been a lot of exploration into the potential treatment of Long COVID by ICWM [11]. With the deepening of research on the treatment of COVID-19 by ICWM, it is necessary to sort out and summarize the published studies.

The growth of coronavirus publications worldwide is improving significantly, especially in most infected countries such as United States, China and Italy [12]. A study has demonstrated and confirmed a massive output in early COVID-19 publications thus far, higher than any previous outbreak and the emergence of COVID-19 has generated an explosive increase in scientific production worldwide across all areas of knowledge [13]. Bibliometric analysis was adopted in this study, which is a quantitative analysis method combining mathematics and statistics. Due to time constraints, the existing bibliometric studies on the treatment of COVID-19 by ICWM are limited in the number and type of literature retrieval [[14], [15], [16]]. As COVID-19-related publications continue to be updated, scientific research will continue to grow with the collaborative efforts of researchers worldwide. Therefore, we use bibliometrics analysis of relevant literature updated in the recent three years to clarify research hotspots and contribute to further research on the treatment of COVID-19.

## 2 Methods

**Fig. 1:**
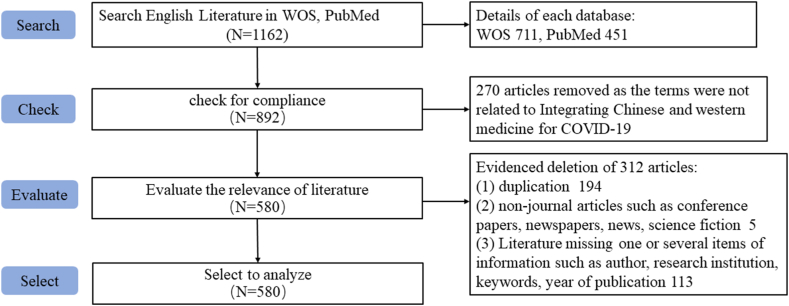
Literature retrieval process of English databases.

**Fig. 2:**
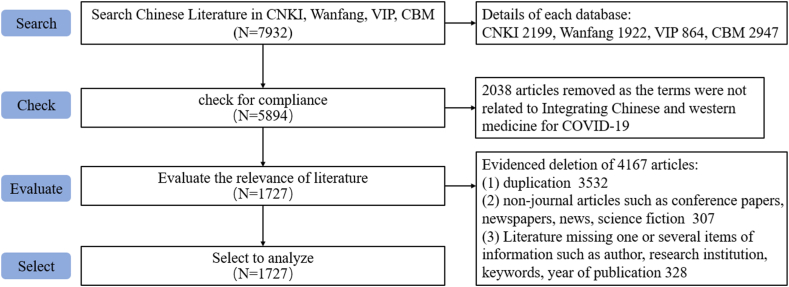
Literature retrieval process of Chinese databases.

Bibliometric methods, are an integral part of research evaluation methodology within the scientific and applied fields and are used increasingly when studying various aspects of science [17]. In this study, the Web of Science core database (WOS), PubMed, China National Knowledge Infrastructure database (CNKI), Wanfang Data Knowledge Service Platform (Wanfang), China Science and Technology Journal Database (VIP), and China Biology Medicine disc (CBM) were searched systematically. All the data from those databases, including information on author names, titles, years, keywords, abstracts, journals, and citations were downloaded, while the CNKI, Wanfang, and VIP databases could not provide citations. All of these data were converted to NoteExpress 3.5.0 to check duplications. After screening, 580 articles in English and 1727 articles in Chinese were retrieved. The process of literature screening was shown in Fig. 1, Fig. 2.

## 3 Search strategies

We downloaded the data from the WOS, PubMed, CNKI, Wanfang, VIP, and CBM on a single day, Oct 31, 2022. The search terms used were the following: (“COVID-19” OR “coronavirus disease 2019” OR “SARS-CoV-2”) AND (“traditional Chinese and Western medicine” OR “integration of traditional and Western medicine” OR “integrative Chinese and Western Medicine” OR “integrative medicine” OR “Combine traditional Chinese and Western medicine” OR “traditional Chinese medicine” OR “herbal medicine”). Only Journal articles written in English and Chinese and published before Oct 31, 2022 were included. The subjects were limited to Medicine and Health.

## 4 Inclusion and exclusion criteria

Inclusion criteria: The topic of the literature is related to the treatment of COVID-19 by ICWM, including but not limited to literature involving ICWM study of COVID-19 in one or more aspects of clinical diagnosis, treatment, prevention, basic experiments, and biological information. All the data were filtered by the exclusion criteria, which were: (1) duplication, (2) non-journal articles such as conference papers, newspapers, news, science fiction, (3) Literature missing one or several items of information such as author, research institution, keywords, year of publication.

## 5 Statistical analysis

In this study, CiteSpace 6.1. R6 and VOSviewer1.6.17 were used to extract the basic characteristics of publications, countries, institutions, keywords, and citations. CiteSpace is a citation visualization analysis software that focuses on the analysis of the potential knowledge contained in the scientific literature. VOSviewer is a program for building and viewing bibliometric maps. Both software can be used to build author or journal maps based on collaborative data, or to build keyword maps based on co-occurrence data [18]. Since Chinese databases could not provide citations, this review was divided into separate discussions for global and domestic databases, in order to show the research gap between China and other countries in the world [19]. Co-occurrence analysis is helpful to quantify common information in various data and reveal the content correlation and common relationship of information [20]. Burst analysis helps to find the trend and vane of research. After searching the literature, the publishing time, journals, countries, institutions, and keywords of the literature were extracted. Then we analyzed the co-occurrence of countries, institutions, keywords, and citations and burst analysis of keywords and citations. The analysis was separated into three parts: (1) a descriptive statistical analysis of the growth pattern, number, year, journals, country, and institution by using CiteSpace and Microsoft Office Excel 2016; (2) a co-occurrence analysis and a burst analysis on the keywords by using VOSviewer, and then (3) a co-citation analysis and a burst analysis to sort out the most influential papers and authors in this field by using CiteSpace.

## 6 Results

### 6.1 Publication growth pattern

**Fig. 3:**
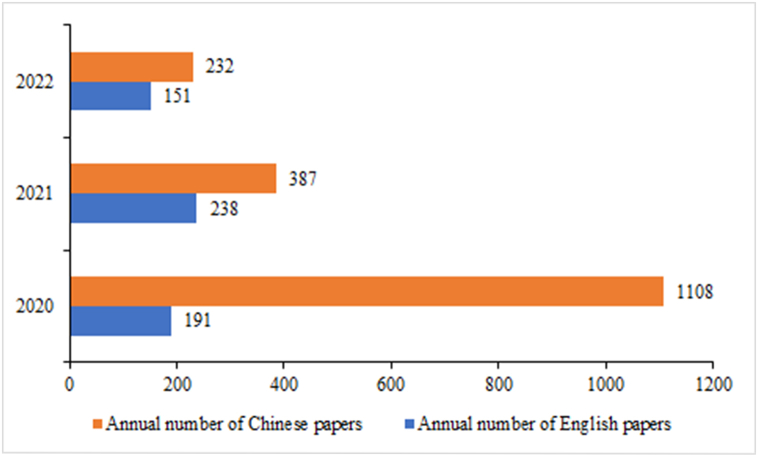
Annual publication volume of research literature on integrated Chinese and Western medicine for COVID-19 during 2020–2022.

To analyze the research hotspots in the field of ICWM for COVID-19, we counted the annual number of English and Chinese publications. As shown in Fig. 3, the development trends in English and Chinese publications are relatively asynchronous. Global attention to ICWM for COVID-19 has grown steadily in the past three years, while the number of research papers in Chinese has been declining since 2020. Due to time limitations, the number of publications in 2022 cannot be fully accounted for, but it will still show a smooth growth trend for the future.

### 6.2 Journal analysis

In total, 580 articles in English were published in 244 journals, among which the three most published journals are *Frontiers in Pharmacology* (44 articles, 7.59%), *Phytomedicine* (25 articles, 4.31%), and *Natural Product Communications* (23 articles, 3.96 %). As it can be seen from Table 1, the articles on ICWM for COVID-19 are relatively diverse in terms of subject coverage. Most of the articles are published in the field of TCM and integrative medicine, but a significant number are published in the field of pharmacology and phytomedicine and other fields.

**Table 1:** Top 10 English journals contributing.

Number	Journal	Documents	Country	Journal Rank	IF
1st	*Frontiers in Pharmacology*	44	Switzerland	Q1	5.988
2nd	*Phytomedicine*	25	Germany	Q1	6.656
3rd	*Natural Product Communications*	23	USA	Q4	1.496
4th	*Journal of Ethnopharmacology*	19	Ireland	Q1	5.195
5th	*American Journal of Chinese Medicine*	15	USA	Q1	6.005
6th	*Chinese Medicine*	10	England	Q1	4.546
7th	*Integrative Medicine Research*	10	South Korea	Q1	4.473
8th	*Pharmacological Research*	9	England	Q1	10.334
9th	*Journal of Integrative Medicine*	9	China	Q2	3.951
10th	*Biomedicine & Pharmacotherapy*	9	France	Q1	7.419

The top 10 Chinese journals are listed in Table 2. All 1727 articles were recorded by 362 journals. In Chinese journals, we referred to the catalog of Chinese Science Citation Database (CSCD) as a standard for evaluating the quality of journals. CSCD includes excellent journals published in many fields in China, which can accurately reflect the academic influence and disciplinary status of journals, so the journals included in CSCD were classified as Core Journals in this study. Among the top 10 Chinese journals, “*Journal of Traditional Chinese Medicine*” published the most papers (62 articles, 3.59%), which was included in CSCD. Other journals that are not listed in the catalog of CSCD have lower publications, which were considered to be of poor influence. Research literature on the treatment of COVID-19 by ICWM is concentrated in a few core journals, and the topic selection of the journals is mainly concentrated in the field of TCM, involving emergency medicine, pharmacology, modern technology and other disciplines. According to the above comparisons, six of ten English journals are from European countries, followed by the United States, while Chinese journals have a lower contribution in this field.

**Table 2:** Top 10 Chinese journals contributing.

Number	Journal	Documents	The Core or Not
1st	*Journal of Traditional Chinese Medicine*	62	Core
2nd	*Tianjin Journal of Traditional Chinese Medicine*	47	Not
3rd	*Chinese Herbal Medicines*	47	Core
4th	*Shanghai Journal of Traditional Chinese Medicine*	45	Not
5th	*Modernization of Traditional Chinese Medicine and Materia Materia-World Science and Technology*	40	Not
6th	*Western Journal of Traditional Chinese Medicine*	28	Not
7th	*Chinese Archives of Traditional Chinese Medicine*	28	Not
8th	*Pharmacology and Clinics of Chinese Materia Medica*	27	Core
9th	*Journal of Emergency in Traditional Chinese Medicine*	25	Not
10th	*Chinese Journal of Experimental Traditional Medical Formulae*	25	Core

### 6.3 Contribution and relationship of countries and institutions

**Fig. 4:**
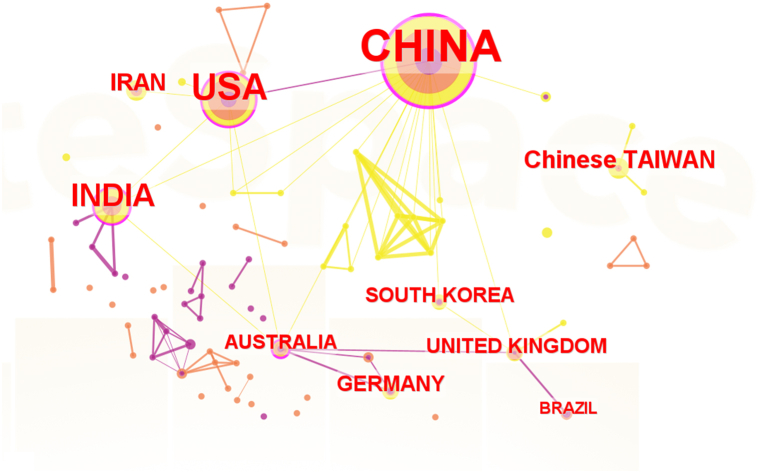
Co-countries analysis of English publications on integrated Chinese and Western medicine for COVID-19 during 2020–2022.

**Fig. 5:**
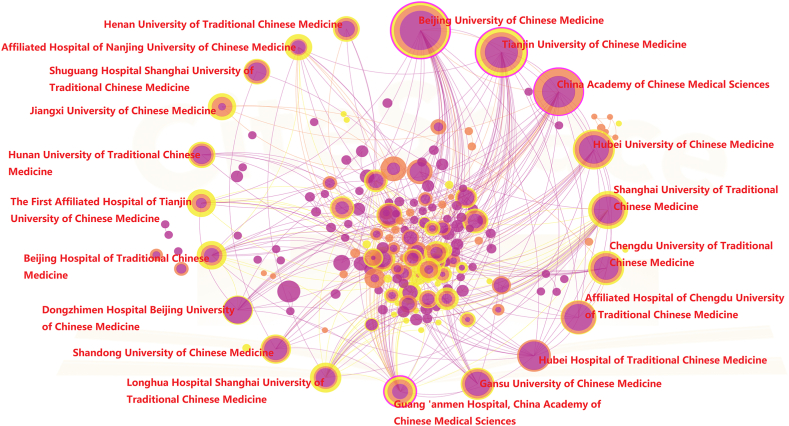
Co-institutions analysis of Chinese publications on integrated Chinese and Western medicine for COVID-19 during 2020–2022.

To identify the distribution and cooperation of countries and institutions, a bibliometric map of research countries and institutions was drawn by CiteSpace (Fig. 4, Fig. 5). In contrast to the journal analysis, China (338 articles, 58.28%) was the most productive region in ICWM for COVID-19, followed by India (51 articles, 8.79%), the USA (47 articles, 8.10%) and the top 10 countries have formed close partnerships. Among them, Asia owned the main research groups. As the major force in the research, China has formed close cooperation respectively with India, the United States, Australia (10 articles, 1.72%), and South Korea (12 articles, 2.06%). Furthermore, we tried to analyze the leading institution in Chinese literature. As shown in Fig. 5, domestic research institutions are mainly universities and hospitals. The most productive institutions were the Beijing University of Chinese Medicine (95 articles, 5.50%), the Tianjin University of Chinese Medicine (71 articles, 4.11%), and the China Academy of Chinese Medical Sciences (63 articles, 3.65%), with a stable cooperative relationship. Among the hospitals, the Affiliated Hospital of Chengdu University of Traditional Chinese Medicine (37 articles, 2.14%), Hubei Hospital of Traditional Chinese Medicine (32 articles, 1.85%), and Guang ‘anmen Hospital of China Academy of Traditional Chinese Medicine (29 articles, 1.68%) are the main medical research institutions. Both universities and hospitals maintain close and stable cooperation in China.

## 7 Co-occurrence analysis of core keywords

**Fig. 6:**
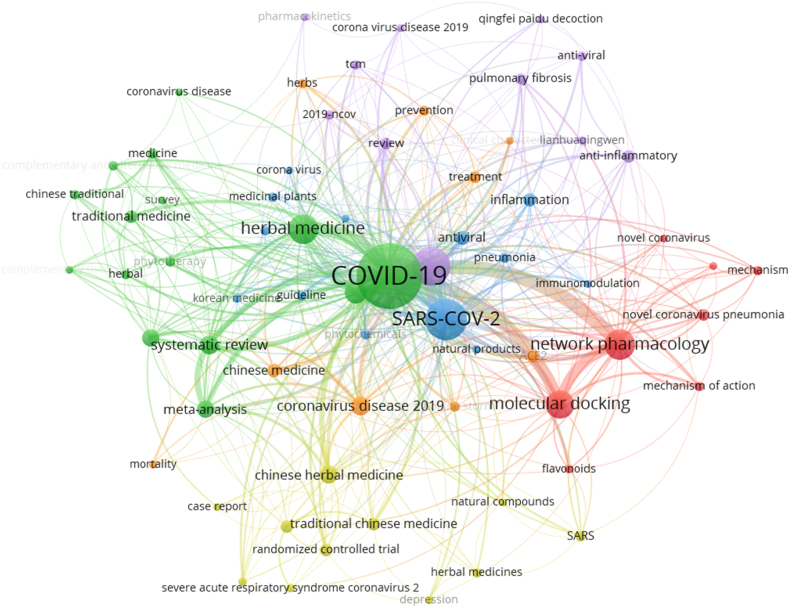
Co-occurring keywords analysis of English literature on integrated Chinese and Western medicine for COVID-19 during 2020–2022.

Keywords are a high generalization of literature research and can accurately reflect the research hotspots of literature. VOSviewer was utilized to construct a knowledge map of co-occurring keywords and keywords clustering. The thickness of the line represents the link strength of the correlation between keywords. In the 580 foreign articles in English, 69 core keywords were selected (minimum number of a term occurrence = 5) (Fig. 6, Table 3). Among them, “COVID-19” (377 times), “SARS-CoV-2” (144 times) and “Traditional Chinese medicine” (124 times) were the most frequent words which are reasonable because they are the search terms. Except for them, other keywords actually reflect hotspots and topics that researchers are focusing on. “Network pharmacology” (78 times),” “molecular docking” (69 times), and “systematic review” (30 times) are the other most frequent words, which showed that the focus of attention in the field of ICWM for COVID-19 is systematic evaluation and network pharmacology.

**Table 3:** Top 20 frequency of core keyword in English articles.

TOP	Keyword	Number	TOP	Keyword	Number
1	COVID-19	377	11	Integrative medicine	26
2	SARS-CoV-2	144	12	Meta-analysis	26
3	Traditional Chinese medicine	124	13	Chinese medicine	15
4	Network pharmacology	78	14	Antiviral	15
5	Herbal medicine	74	15	inflammation	15
6	molecular docking	69	16	Ayurveda	14
7	Coronavirus	42	17	Traditional medicine	14
8	Coronavirus disease 2019	31	18	Randomized controlled trial	13
9	systematic review	30	19	Anti-inflammatory	13
10	Chinese Herbal Medicine	27	20	ACE2	11

The core keywords were divided into six clusters by color (Fig. 6). The green group mainly involved systematic reviews and meta-analyses of the effectiveness of integrative medicine or phytotherapy. The red group which was far from the green group, focused on network pharmacology and molecular docking, including a study on the anti-inflammatory mechanism of Chinese herbal ingredients, such as flavonoids. The blue group, focused on the SARS-CoV-2, located in the center of the cluster, mainly involved the antiviral effects of medicinal phytochemicals and immunomodulation of the body. There was a thick line between the red group and the green group, indicating a close connection between the two groups. Randomized controlled trials are also one of the major types of studies on the treatment of COVID-19 with ICWM, which can be seen from the yellow group. The rest purple and orange groups respectively focused on *Qingfei Paidu Decoction* and prevention and treatment of COVID-19.

**Fig. 7:**
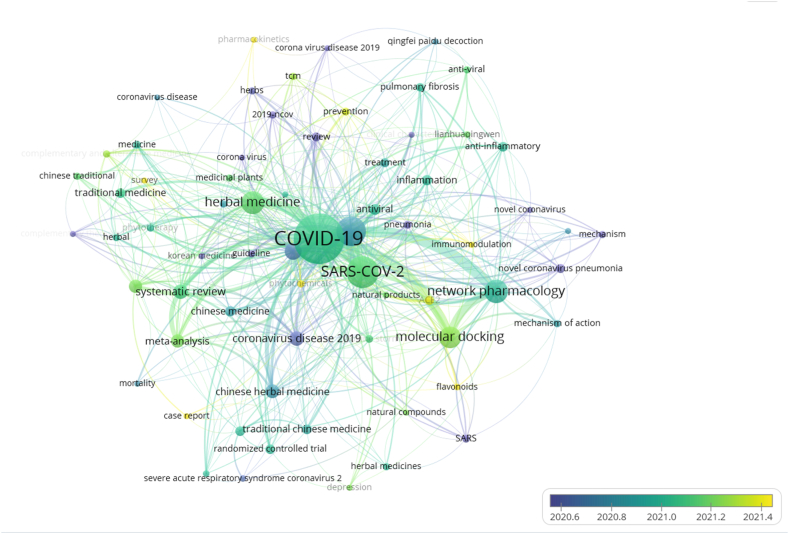
Burst keywords analysis of English literature on integrated Chinese and Western medicine for COVID-19 during 2020–2022.

Moreover, to analyze the frontier topics, we collected the burst keywords (Fig. 7). Unlike the above, Fig. 7 showed each keyword with an average publication year in the research field. The yellower the node color, the later the keyword appears; the bluer the node color, the longer the keyword appears. As a whole, the color distribution showed a trend from blue-purple in the center to yellow-green around the edges, which means the topics have gradually focused on prevention and pharmacokinetics in recent years.

In contrast, 92 core keywords were screened in 1727 articles in Chinese (minimum number of a term occurrence = 10) and the top 20 high-frequency keywords are sorted in Table 4. Excluded search terms, “Network pharmacology” (98 times), “pestilence” (94 times), and “*Qingfei Paidu Decoction*” (70 times) were the top three hot words. In addition, characteristics of TCM therapy such as syndrome differentiation and clinical classification of COVID-19, such as severe and convalescing stage, are also hot topics for domestic scholars.

**Table 4:** Top 20 frequency of core keyword in Chinese articles.

TOP	Keyword	Number	TOP	Keyword	Number
1	COVID-19	1434	11	Chinese medicine	52
2	Traditional Chinese medicine	374	12	pneumonia	52
3	novel coronavirus	241	13	Integrated treatment of Chinese and Western medicine	50
4	Integration of traditional Chinese and Western medicine	146	14	Diagnosis and treatment plan	49
5	Network pharmacology	98	15	Convalescence	43
6	pestilence	94	16	Integrated Chinese and Western medicine therapy	37
7	*Qingfei Paidu Decoction*	70	17	prevent	36
8	Treatment based on syndrome differentiation	57	18	Expert consensus	35
9	TCM therapy	55	19	Severe type	33
10	Molecular docking	53	20	Prevention and treatment of disease	31

**Fig. 8:**
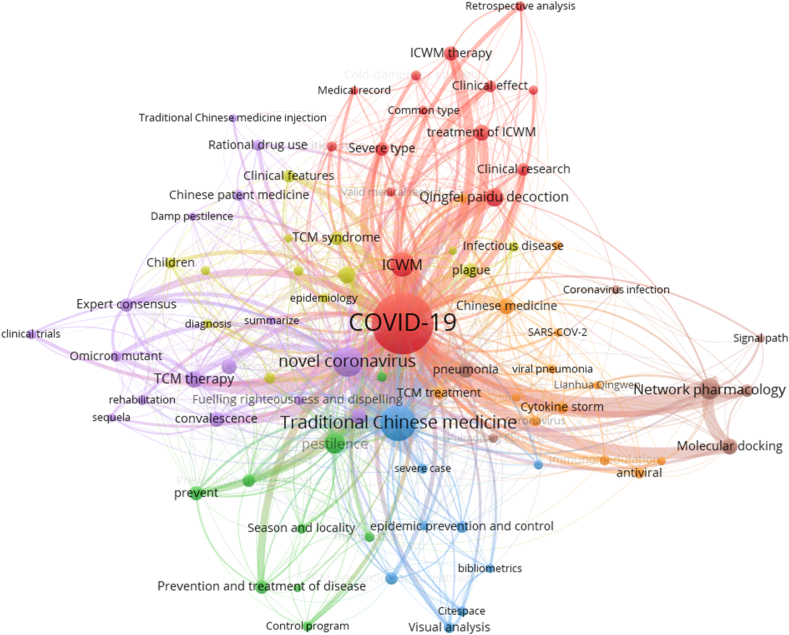
Co-occurring keywords analysis of Chinese literature on integrated Chinese and Western medicine for COVID-19 during 2020–2022.

A total of 7 clusters were obtained by cluster analysis of core keywords in Chinese research literature (Fig. 8). It can be seen that the clusters in the Chinese literature are closely related, especially the red group, green group, and orange group. All of them described the clinical effectiveness of a variety of integrated Chinese and Western therapies for different clinical stages of COVID-19. The purple group was related to expert consensus on TCM therapy in the convalescence of COVID-19, and the rational drug use of Chinese patent medicine and traditional Chinese medicine injection. From the perspective of bibliometrics, the blue group discussed the diagnosis and treatment plan of the novel coronavirus pneumonia and the characteristics of TCM prevention and treatment. The yellow group elucidated the understanding of the epidemic spread and clinical features of the novel coronavirus pneumonia and the treatment based on syndrome differentiation from the understanding of plague. The brown group explored the mechanism of the treatment of the novel coronavirus pneumonia with TCM prescriptions by network pharmacology, molecular docking and other methods. Chinese literature focused on clinical studies of ICWM in the treatment of COVID-19, such as retrospective studies, clinical features and TCM syndrome analysis, while English literature focused on therapeutic mechanism studies and evidence-based medicine studies, such as systematic review and meta-analysis. Compared with English literature, Chinese literature covered richer content, which may be related to the extensive application of ICWM in China.

**Fig. 9:**
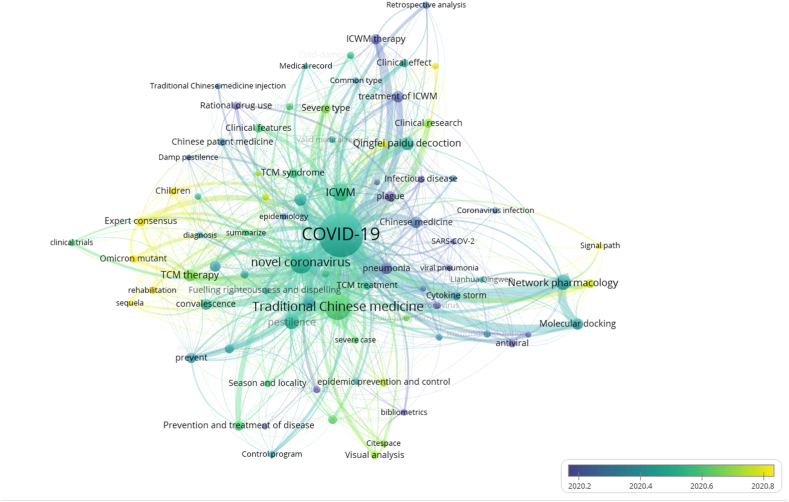
Burst keywords analysis of Chinese literature on integrated Chinese and Western medicine for COVID-19 during 2020–2022.

The burst keywords analysis in Fig. 9 also showed the change in research hotspots from 2020 to 2022. The color in the figure changes from blue-green in the middle to light yellow in the periphery, which means that the focus of Chinese research literature on the treatment of COVID-19 by ICWM has gradually changed from clinical trial research to efficacy evaluation and the study on the mechanism of action and signal path of related drugs. Especially in the past two years, domestic researchers have made more explorations in the aspects of expert consensus, rehabilitation of sequela and action targets of TCM prescription.

### 7.1 Co-citation reference analysis

**Fig. 10:**
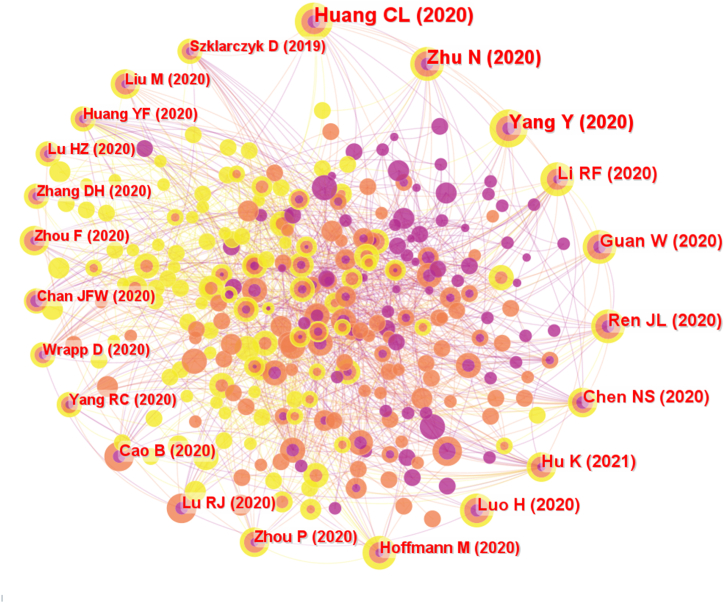
Co-citation reference analysis of English literature on integrated Chinese and Western medicine for COVID-19 during 2020–2022.

Co-citation refers to the situation that two or more articles are cited by other articles at the same time, and the two articles are considered to be a co-citation relationship [18]. To better understand the research literature's influence and connection in the field, the top 10 most cited papers were identified and are listed in Table 5. However, the Web of Science core database and PubMed are the research targets because of the limitation of Chinese databases. We screened the highly cited papers that had been cited more than 20 times (Fig. 10). Among them, the article by Huang CL published in the *Lancet* in 2020 was the most cited so far (81 citations). It reported the epidemiological, clinical, laboratory, and radiological characteristics and treatment and clinical outcomes of patients infected with the 2019 novel coronavirus in Wuhan, China [21]. Most of the highly cited papers in this field are related to epidemiological research on COVID-19, clinical research on the treatment of COVID-19 by TCM, and molecular biology research on COVID-19. These papers have been highly cited in the “introduction” section of other papers, trying to provide new ideas for summarizing the effectiveness of ICWM in the treatment of COVID-19 and exploring its therapeutic mechanism.

**Table 5:** TOP 10 highly cited papers in English.

TOP	Title	Year	Number of cited	Journal
1st	Clinical features of patients infected with 2019 novel coronavirus in Wuhan, China	2020	81	*LANCET*
2nd	A Novel Coronavirus from Patients with Pneumonia in China, 2019	2020	61	*NEW ENGL J MED*
3rd	Traditional Chinese Medicine in the Treatment of Patients Infected with 2019-New Coronavirus (SARS-CoV-2): A Review and Perspective	2020	55	*INT J BIOL SCI*
4th	Lianhuaqingwen exerts anti-viral and anti-inflammatory activity against novel coronavirus (SARS-CoV-2)	2020	54	*PHARMACOL RES*
5th	Clinical Characteristics of Coronavirus Disease 2019 in China	2020	49	*NEW ENGL J MED*
6th	Traditional Chinese medicine for COVID-19 treatment	2020	46	*PHARMACOL RES*
7th	Efficacy and safety of Lianhuaqingwen capsules, a repurposed Chinese herb, in patients with coronavirus disease 2019: A multicenter, prospective, randomized controlled trial	2020	40	*PHYTOMEDICINE*
8th	Epidemiological and clinical characteristics of 99 cases of 2019 novel coronavirus pneumonia in Wuhan, China: a descriptive study	2021	40	*LANCET*
9th	Can Chinese Medicine Be Used for Prevention of Corona Virus Disease 2019 (COVID-19)? A Review of Historical Classics, Research Evidence and Current Prevention Programs	2020	34	*CHIN J INTEGR MED*
10th	SARS-CoV-2 Cell Entry Depends on ACE2 and TMPRSS2 and Is Blocked by a Clinically Proven Protease Inhibitor	2020	33	*CELL*

**Fig. 11:**
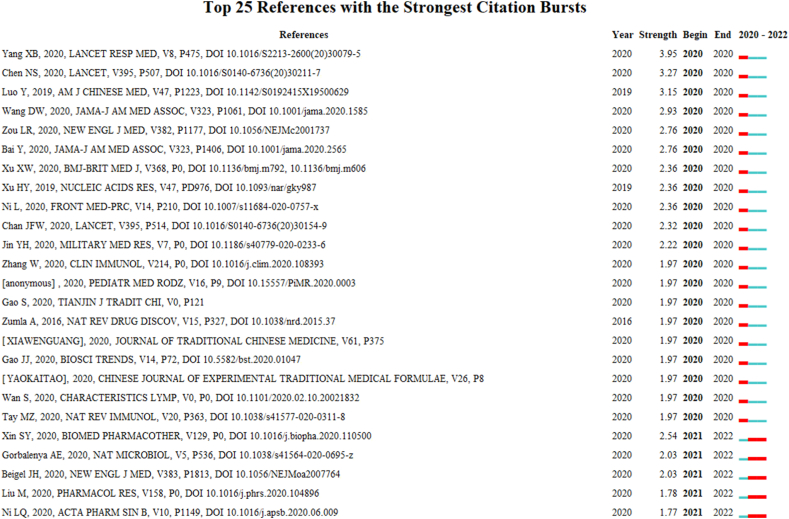
Burst analysis of co-citation reference of English literature on integrated Chinese and Western medicine for COVID-19 during 2020–2022.

Fig. 11 showed the top 25 references with the strongest citation bursts. After reading these literature one by one, we found that from the initial stage of the outbreak in 2020–2022, the industry's focus on COVID-19 ranged from clinical characteristics analysis to the formation of diagnosis and treatment guidelines, drug discovery, and the evaluation of the effectiveness of ICWM. It can be seen that the effectiveness and safety of ICWM for COVID-19 have been confirmed [8,22], and the treatment of ICWM for COVID-19 has attracted the attention of scholars around the world and is still widely popular. It implies that the research related to ICWM may continue to explode in the future.

## 8 Discussion

Bibliometrics plays an important role in ranking the performance of researchers, institutions, or journals within the international arena in a specified field [23]. In this study, two research databases were established to analyze the current situation and development trend of the research on ICWM for COVID-19 at home and abroad from 2020 to 2022, and determine the contributions of different countries and institutions and their cooperative relationships, especially those of domestic institutions in China. At the same time, cluster keywords mapping and burst terms analysis were used to track research hotspots and frontiers. Finally, co-citation analysis was used in the WOS and PubMed databases to analyze the influence and connections of the cited literature in the field to encourage more dialogue among scholars. By comparing the differences in research topics, trends and hotspots, the gap among countries was highlighted, and future research directions and opportunities were revealed.

In terms of publication years, the gradual and slow publication trend of Chinese and English literature is basically the same. As the ICWM has been widely used in China and even the world, relevant research has become more in-depth. In the past three years, the number of global publications on the treatment of COVID-19 by ICWM was the largest in 2020, indicating that the treatment of ICWM has attracted the attention of scholars since the outbreak of COVID-19 and is expected to continue to maintain a steady growth trend. It underlies an unmet need for solutions against COVID-19 pandemic. From the perspective of journals, the journals with the greatest contribution to this field are mainly those related to ICWM, as well as pharmacology, biomedicine, and other fields, which means that the treatment of COVID-19 by ICWM at home and abroad has different emphases, involving the intersection of multiple fields. Compared with English journals, although Chinese journals are about 1.5 times as large as English journals in quantity, Chinese journals have less influence on quality. As for future developments, it is likely to be showcased again in the open journals of *Frontiers in Pharmacology* and *Phytomedicine*, which have published the most articles in the past. In addition, China is the leading country in ICWM research, followed by India and the United States. Asia has stable cooperative relations with North America and Europe. The domestic institutions could become potential to be partners committed to this area. It is expected that major top research institutions will remain in China in the future due to the large amount of funding guaranteed in this field. On the other hand, through co-citation analysis, the paper published by Huang CL in the *Lancet* in 2020 was the most cited paper so far, which laid the foundation for clinical research on COVID-19 and greatly promoted the development of this research field. As shown in Fig. 10, the most cited literature was based on the basic epidemiological investigation (such as clinical characteristics) of COVID-19 and the effectiveness of TCM treatment, which laid a solid foundation for the prevention and treatment of COVID-19 by ICWM in the later period, as well as the relevant mechanism of action.

From the analysis of research hotspots, global hot research topics mainly focused on the effectiveness evaluation of ICWM in the treatment of COVID-19 and the mechanism of action of TCM therapy, and then gradually expanded to the field of ICWM in the prevention of COVID-19. The analysis of keywords in China showed that clinical effectiveness, diagnosis, and treatment guidelines of different disease stages are still the main research directions of ICWM in the treatment of COVID-19, especially the application of syndrome differentiation of TCM in the convalescence period of COVID-19. Some domestic studies have also used network pharmacology, bibliometrics, and other methods to analyze the targets of TCM prescriptions in the treatment of COVID-19 and the research status of COVID-19.

A comparison of keywords in Chinese and English literature showed that both of them paid attention to network pharmacological research, *Qingfei Paidu Decoction* (QFPDD). Network pharmacology and molecular docking techniques have been widely used to establish substance-based intervention networks to explore the targets, signaling pathways, and mechanisms of action of TCM prescriptions against COVID-19 [[24], [25], [26]]. Multiple network pharmacological and molecular docking studies have shown that herbal medicines show a high affinity for ACE2, the critical functional receptor for SARS-CoV-2, in the treatment of COVID-19 [27], including formulas (such as *Xuebijing Injection*, *Shufeng Jiedu Capsule*, *Huashi Baidu Formula*, and *Maxing Shigan Decoction*) [[27], [28], [29], [30], [31]] and active ingredients (such as quercetin, glycyrrhizic acid, and salvianolic acids) [[32], [33], [34]]. QFPDD, as a multi-component herbal formula, has been recommended for the clinical treatment of COVID-19 by many provinces throughout China [35,36]. According to a clinical retrospective study, QFPDD combined with Western medicine demonstrated significant anti-inflammatory effects and tended to mitigate the extent of multi-organ impairment compared with those of only Western medicine in patients with mild and moderate COVID-19 [37]. Systems pharmacological studies have shown that it could exhibit immune regulation, anti-infection, anti-inflammation, and metabolic disorder to perform a corresponding therapeutic effect by regulating a complex molecular network with safety and efficacy [[38], [39], [40]]. Compared with English literature, the number of literature and the types of prescriptions in Chinese Internet pharmacology studies are more abundant. However, the influence factors in Chinese literature are not high, mainly due to the low research innovation, simple research methods, and lack of experimental verification.

Currently, from the perspective of research trend, the future research types in Chinese and English will continue to be inclined to the direction of mechanism of action, and gradually transit from conventional treatment of COVID-19 to convalescent treatment and prevention of COVID-19 with ICWM. In the convalescence treatment of COVID-19, the combination of Western medicine's multidisciplinary rehabilitation mode with TCM decoction, acupuncture and massage, traditional exercises and other methods can promote the rehabilitation process, effectively improve the sequelae of COVID-19 and improve the quality of life [41,42]. A study has shown that medical staff can prevent iatrogenic infections by taking herbal decoctions and moxibustion based on TCM principles in the hospital [43]. Prevention methods of ICWM have been widely used in community prevention, home isolation, and other situations, thus reducing the risk of transmission and protecting public health [[44], [45], [46]]. With the development of the global epidemic and the increase of discharged patients, the integration of traditional Chinese and Western medicine to prevent and control COVID-19 and the treatment of the recovery period will be the focus of future research. Although domestic and global cooperation is not close at present, with the convergence of research trends in the future and the strong support of Chinese funding, it is expected that more Chinese researchers will have a higher quality of research output in this field.

On May 5, 2023, the World Health Organization declared that COVID-19 as a global health emergency had got an end, however, this does not mean the pandemic is over as a global health threat [47]. There are many research challenges and many open questions about the sequelae of COVID-19, particularly relating to effective treatments [48]. ICWM has played an important role in improving the clinical symptoms of COVID-19 and reducing the incidence of critical illness and mortality. More importantly, ICWM may be a key to further promoting rehabilitation and resolution of residual symptoms [49]. However, the integration of Chinese and Western medicine itself has some limitations, and these problems are also present in the application of ICWM in COVID-19 due to the integration of Chinese medicine in Western health systems and research pending a profound evaluation of effectiveness and security, the lack of science-based conceptualization and standardization of the diagnostic and therapeutic processes of Chinese medicine [50], and the lack of molecular mechanisms of Chinese medicine [51]. In the future, high-quality evidence were still needed to justify the effectiveness of ICWM for the pandemic, and the potential of TCM and ICWM for treating long COVID or post-COVID conditions was worth exploring.

## 9 Limitation

Due to the limitation of databases, the co-citation of Chinese literature was not analyzed. As a result, high-quality Chinese articles that made important contributions to this field cannot be collated or compared with English articles. Moreover, this literature only selected two English databases and four Chinese databases to represent the current research status in China and abroad, and did not consider the publication bias of the literature, and some literature may be ignored. In the future, relevant research can further expand the scope of the literature search and more comprehensively analyze current research hotspots and fronts.

## 10 Conclusions

Through the analysis and comparison of Chinese and English research literature databases, it can be seen that the treatment of COVID-19 by ICWM has become a worldwide research hotspot. Both Chinese and English literature focuses on network pharmacology and molecular docking techniques as well as *Qingfei Paidu Decoction*. Although there are differences in the specific contents between Chinese and English publications, the development trend of research types to the mechanism of action, and the development trend of research contents to the recovery period treatment and the prevention of COVID-19 by ICWM are consistent.

## Financial support

This work was supported by the High-level traditional Chinese medicine key subjects construction project of National Administration of Traditional Chinese Medicine——Evidence-based Traditional Chinese Medicine (zyyzdxk-2023249) and Fundamental Research Funds for the Central Universities (2022-JYB-PY-013).

## Data availability statement

All data generated or analyzed during this study are included in this article. Further information could be acquired by contacting with the corresponding author.

## CRediT authorship contribution statement

**Meijiao Du:** Writing – original draft, Formal analysis. **Hongkai Li:** Writing – review & editing, Software, Formal analysis. **Huijuan Guo:** Writing – review & editing. **Xiaowen Zhang:** Writing – review & editing, Visualization, Software, Formal analysis. **Hongguo Rong:** Writing – review & editing, Supervision, Funding acquisition, Data curation, Conceptualization. **Xuezeng Hao:** Writing – review & editing, Project administration, Data curation, Conceptualization.

## Declaration of competing interest

The authors declare that they have no competing interests.
